# Ecological effects of dams, alien fish, and physiochemical environmental factors on homogeneity/heterogeneity of fish community in four tributaries of the Pearl River in China

**DOI:** 10.1002/ece3.2920

**Published:** 2017-04-22

**Authors:** Lei Zeng, Lei Zhou, Ding‐Li Guo, Dong‐Hua Fu, Peng Xu, Shuang Zeng, Qin‐Dong Tang, An‐Luo Chen, Fei‐Qiao Chen, Yong Luo, Gui‐Feng Li

**Affiliations:** ^1^ Guangdong Province Key Laboratory for Aquatic Economic AnimalsSchool of Life SciencesSun Yat‐Sen UniversityGuangzhouChina; ^2^ South China Sea Fisheries Research InstituteChinese Academy of Fishery SciencesGuangzhouChina; ^3^ School of Life SciencesInstitute of Aquatic Economic AnimalsSun Yat‐Sen UniversityGuangzhouChina; ^4^ South China Sea Bio‐Resource Exploitation and Utilization Collaborative Innovation CenterSun Yat‐Sen UniversityGuangzhouChina; ^5^ College of Life ScienceSouth China Agricultural UniversityGuangzhouChina; ^6^ Fishery, Animal Husbandry and Veterinary Bureau of Tianyang CountyBaiseChina

**Keywords:** biotic/abiotic factors, canonical correspondence analysis, fish community, homogenous/heterogeneous trend

## Abstract

In this study, we aimed to characterize the fish community structure and identify the drivers contributing to homogenization/differentiation processes in four tributaries to the Pearl River, Guangxi Province, China, over the past few decades. We sampled 22 sites seasonally from 2013 through 2015, and these sites were selected based on archived records of previous sampling conducted in the 1980s. Jaccard's faunal similarity index, cluster analysis, and canonical correspondence analysis (CCA) were applied to describe the homogenization/differentiation of fish community and illustrate the potential effectors. The number of fish species present in three of the four sampled tributaries declined dramatically over the past 30 years, leading toward a trend of increased fish community homogeneity throughout the watershed. Results from multidimensional scaling and cluster analyses allowed us to divide the study area into two distinct ecoregions. Four species (*yellow catfish Pelteobagrus fulvidraco*,* pond loach Misgurnus anguillicaudatus*,* Nile tilapia Oreochromis niloticus*, and *sharpbelly Hemiculter leucisculus*) were considered to be indicative fish species contributing more than 5% of the dissimilarity between the two eco‐regions according to the results of similarity percentage procedure. Results from CCA revealed that pH and latitude corresponded with the dominant fish species of each respective tributary. More specifically, CCA results allowed us to classify dominant fish species into three distinct groups. The first group was mainly located in Guijiang characterized by higher latitudes and lower pH values, the second group was widespread in the four tributaries, and the last group was primarily distributed in Yujiang, Youjiang, and Zuojiang characterized by lower latitudes and higher pH values. Spatial differentiation of fish community structure and temporal homogeneity of species composition were attributed to the joint actions of human interventions including construction of dams and introductions of exotic fish species that led to habitat degeneration and fragmentation, and unequal interspecies competitions.

## 1 Introduction

Freshwater ecosystems are of prominent global importance, as over 10,000 fish species (c. 40% of global fish diversity) live in freshwater (Lundberg & Gill, 2000), which accounts for only 2.5% of global water resources. The biodiversity of any given fish community is often used as a bioindicator for the assessment of water quality, river network connectivity, or flow regime (Fausch, Lyons, Karr, & Angermeier, 1990; McCormick & Larsen, 2000). Freshwater fishes represent some of the most threatened fauna in the world, owing to threats from anthropogenic factors which can be grouped under four interacting categories: overexploitation, water pollution, dam construction, and alien introduction. The combined influences of these anthropogenic factors upon fish biodiversity have been a hot topic worldwide (Allan & Flecker, 1993; Jackson & Running, 2001; Malmqvist & Rundle, 2002; Naiman & Turner, 2000; Rahel, 2003). Knowledge of anthropogenic threats is increasing among the scientific community, and a series of analogous research programs are in operation around the world (Brown, 2000; Dudgeon, 2000). However, many inventories of fish biodiversity in freshwater are distinctly insufficient in many parts of the world, and rates of species loss may be higher than currently estimated. Thus, wider dissemination and emphasis on the issue of fish biodiversity is urgently needed.

However, a growing consensus has been reached in recent decades among ecologists that studying biodiversity patterns on account of species occurrence and abundance will not be sufficient to explain a few ecological phenomena, including community structure dynamic, multispecies interactions, and determinants of species’ distributions (Wellborn, Skelly, & Werner, 1996). Towards these challenges, multidimensional approaches have been initiated to be used with increasing effectiveness and frequency to characterizing diversity within landscapes. Geographical variation across communities can be quantified by β‐diversity that identified another significant aspect of biological complexity (Cadotte, 2011; Crist & Veech, 2006; Loreau, 2000). At the tributary scope, β‐diversity assessment may be performed by comparing matrices of pairwise community distances (Bray & Curtis, 1957) versus Euclidean distances. The spatial differentiation significance of fish communities within tributaries is then evaluated by the Mantel test (Fortin & Gurevitch, 2001). If historical fish community data are available, a comparison between old and new spatial patterns of fish community may indicate whether changes between them occurred and, indirectly, shed light on which human and/or natural factors are responsible for the changes.

Quantifying the biodiversity of fish communities and the associated human management of their habitats are particularly challenging in China, mostly due to dam construction and the introduction of alien fish species. There have been more than 96,000 of dams constructed since the 1950s, leading to losses of fish species, decreases in community diversity, and habitat destruction/defragmentation, either directly or indirectly (Fu, Wu, Chen, Wu, & Lei, 2003; Liu, 1984; Zeng, 1990). Published scientific literature is replete with studies investigating the effects of hydroprojects (Dudgeon, 2000; Dynesius & Nilsson, 1994; He, Li, & Zhang, 2007). Construction of cascade dams breaks the seasonal migration paths of migratory fishes which contribute to the heterogeneity of biota (Freeman et al., 2002). The operation of these dams will cause environmental variations to some extent, including flow modification, depth, dissolved oxygen, and temperature fluctuation (Liu, 1992; Salazar, 2000). Furthermore, dams will simplify the physical structure of the natural watercourse with the loss of heterogeneous habitats such as lakes and riffles. Biodiversity changes resulting from dam construction may increase susceptibility to species invasion in a number of ways (Chapin et al., 2000). Human introductions of alien fish species are deemed to be the second largest cause of species extinction after habitat destruction (Vitousek, Mooney, Lubchenco, & Melillo, 1997; Xie, Li, Gregg, & Li, 2001). An impressive amount of autochthonous species have been extirpated throughout the world following the introduction of alien fishes into their habitats (Lever, 1996; Taylor, Courtenay, & McCann, 1984). Exotic fish species can affect local ecosystems in diverse ways (Simon & Townsend, 2003). They can prey on or compete with native fish for food and other resources, which may lead to dramatic declines in the abundance of some local species, and even the extinction of some species in extreme circumstances (Dudgeon & Smith, 2006; Raghavan, Prasad, Anvar‐Ali, & Pereira, 2008; Zhang, Cao, & Chen, 1997). Introductions of alien fish and extirpations of native fish in freshwater may alter fish community dynamics in a given space or over a length of time, thus resulting with overall community homogeneity (McKinney & Lockwood, 1999; Olden, 2006) or heterogeneity (Taylor, 2010). However, despite the adverse effects of alien fish, they have been introduced into many regions in large numbers, both unintentionally and intentionally, for various reasons (Welcomme, 1988). Aquaculture was the main motivation for introduction in 38.7% of the FAO Database records on Introductions of Aquatic Species, and half of the 1205 records for aquaculture programs are reported to have allowed escapees to become established in the wild (http://www.fao.org/fishery/dias/en). For instance, *Nile tilapia* was a typical alien species introduced in China for industrialized aquaculture in the 1950s and has become firmly established in the Pearl River at present, which severely threatened the survival of native species, as well as the structure and function of the freshwater ecosystem (Tan, 2012).

The scarcity of available data on the structure and distribution of fish species in space and time greatly limits the formulation of current biodiversity conservation strategies in the Pearl River, China. Thus, conducting a comprehensive assessment of fish biodiversity and the variation of fish communities in the spatial–temporal scale of the branches of the Pearl River in China is urgently needed.

## 2 Methods

### 2.1 Study area

**Figure 1:**
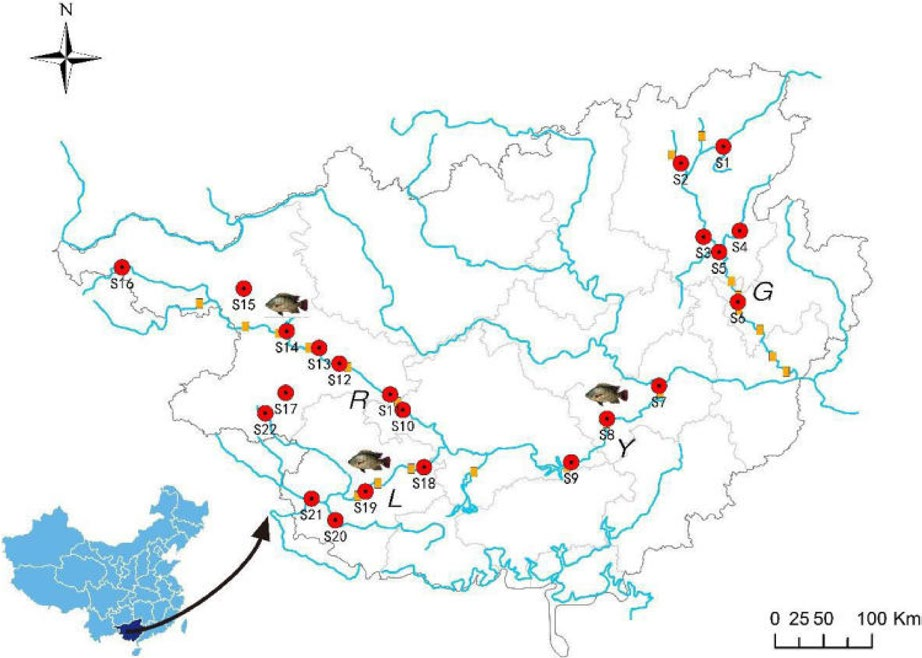
Map of the study area showing the 22 sampling sites (from S1 to S22) distributed along the four tributaries (G, Y, R, L) in Guangxi Province, China, in 2014 and 2015. G, Y, R, and L represent Guijiang, Yujiang, Youjiang, and Zuojiang, respectively. Tributaries labeled with photos were *Tilapia* aquaculture areas in the studied areas. Yellow rectangles exhibit the large/medium dams (volume capacity > 1 billion m³) in each tributary

Guijiang (G), Yujiang (Y), Youjiang (R), and Zuojiang (L) are four major tributaries of the Xijiang River—which is the main stream of the Pearl River in China. Guijiang flows southwestward and Yujiang flows in the opposite direction to join the Xijiang River at Guangxi Province to form a large watershed area lying between 23.47°N and 23.40°N, and 111.31°E and 110.10°E (Figure 1). Guijiang is 438 km long with a catchment area of about 19,288 km^2^. Yujiang, Youjiang, and Zuojiang have a joint length of 1,145 km and a catchment area of 90,656 km^2^ (Lu, 1990). We established 22 sample sites (S1–S22) within the four tributaries (G, Y, R, and L) corresponding to sites investigated by Lu et al. during the 1980s. The distance between these sites differed, as the watercourses were anfractuous. The proliferation of dam construction and development of aquaculture, especially farming of nonnative fish species, have impacted native freshwater fauna. The studied tributaries (G, Y, R, and L) are fragmented by 7, 3, 6, and 3 cascade medium‐/large‐size dams (volume capacity larger than one billion cubic meter), respectively, and these tributaries contain hundreds of smaller unnamed dams and barriers according to China census for water in 2012 (Figure 1). *Nile tilapia* was one of the exotic species introduced for aquaculture in the Pearl River. Contemporary *N. tilapia* aquaculture facilities are ubiquitous throughout the Guangxi Province, and as a result of containment failures, natural populations have become widely established in the Pearl River (Figure 1; Tan, 2012).

### 2.2 Sample procedure

Seasonal data collections were performed in each sample site in November 2013 and March, May, August, and November in 2014, and March, May, and August in 2015 through local veteran fishermen. Multiple fishing methods including cast nets, gill nets (with various mesh sizes), and cage nets were combined to optimize sampling effort, minimize the bias due to specific gears, and maximize the representation of fish populations in each sample site. Captured fish were sorted and identified to species and weighed to the nearest gram. Species‐specific abundances and biomass estimates from each sample were standardized by the number of individuals and kg per unit of effort, respectively (e.g., kg day^−1^ vessel^−1^). We also visited fish markets and landing centers near the sample sites to look for the presence of any species that were not represented in our experimental sampling procedures. We found that 95% of the fish species in fish markets and landing centers were captured in our sampling, therefore indicating that our sampling procedures provided adequate representation of site‐specific fish communities.

Additional physiochemical data including water level, pH, DO, NH_3_–N, MnO_4_^−^, and total phosphorus (TP) of the investigated tributaries in 2014 were retrieved from the Hydrology and Water Resources Bureau of Guangxi Zhuang Autonomous Region. The latitude of each sample site was obtained in the field by a global position system GARMIN *eTrex*. Historical fish community and physiochemical data were abstracted from an investigation of the Pearl River in the 1980s (Lu, 1990).

### 2.3 Data process and analysis

To analyze the temporal variations of species composition and distribution in the four tributaries over the past few decades, we compared data gathered for this study with data from studies conducted during the 1980s. Due to the lack of standardized estimates of abundance from previous data for most sample sites, we restricted our comparisons to measures based on presence/absence data by calculating Jaccard's coefficient of faunal similarity in time and space.

(1) $$S_{j}=j/a+b-j,$$

where *S*_*j*_ is the similarity between any pairwise tributaries and *j* is the number of common species in both tributaries. The value of *a* is the total number of species in the first tributary, and *b* is the total number of species in the second tributary.

Margalef species richness (*d*) and Shannon–Wiener diversity (*H*′) were selected in this study to evaluate fish diversity based on biomass data in each tributary. Diversity indices calculated on biomass data can express the distribution of biological energy among fish species more precisely, as the individual size between the same or different fish species may vary significantly (Wilhm, 1968). The spatial variations of *d*,* H*′, and physiochemical environmental factors were analyzed with one‐way ANOVA respectively, and the correlation among those factors was analyzed by Pearson correlation analysis.

β‐Diversity was introduced to visualize the distinctions of site‐specific fish communities in terms of tributaries and seasons. Cluster and ordination methods were performed based on the Bray–Curtis similarity matrix of fish biomass data. A square root transformation was applied to the similarity matrix to minimize the influence of dominants. Analysis of similarities (ANOSIM) was carried out according to the results from cluster analysis to test for inner‐ and intergroup (dis)similarity (Primer 5; Clarke & Gorley, 2001). Similarity percentage procedure (SIMPER) on transformed variables was used to identify the species that contributed most to the inner‐group similarity and intergroup dissimilarity (Primer 5; Clarke & Gorley, 2001). Nonparametric multidimensional scaling (MDS) was used to visualize the distribution of indicative species in each group, according to the results of a similarity percentage procedure. Species contributing to the differences among groups greater than 5% were considered to be the grouping indicators (Zhang, 2007).

Canonical correspondence analysis (CCA), designed for direct analysis of relationships between multivariate ecological data (Braak, 1986), was applied to physiochemical data from thirteen sample sites along with the corresponding dominant fish populations (standardized using log_10_(*X* + 1) transformation). Statistical significance of the CCA relationship between the set of physiochemical data and fish species was evaluated using a Monte Carlo permutation test with 999 unrestricted permutations.

The graphs in this article were constructed using Origin 8.0 and ArcView GIS software, and statistical analyses were performed using SPSS 21.0. The spatial and temporal variation of fish community structure was processed using Primer 5, and the CCA was processed using Canoco 4.5.

## 3 Results

### 3.1 Species composition and similarity analysis

**Figure 2:**
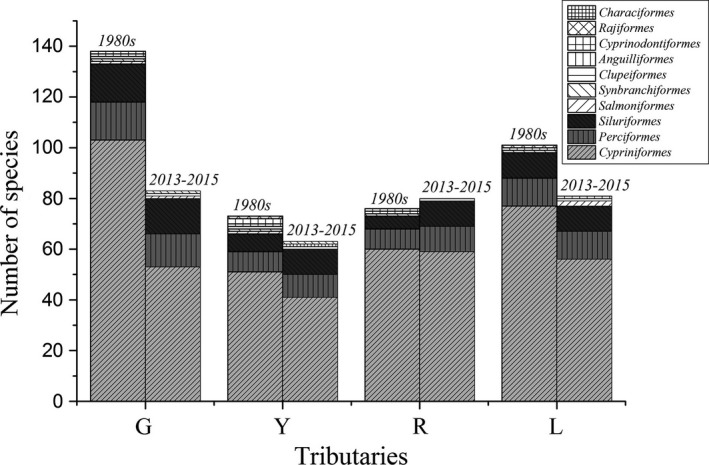
Comparison of the number of fish species in order taxonomy level in four tributaries (G, Y, R, and L represent Guijiang, Yujiang, Youjiang, and Zuojiang respectively) of different time periods

A total of 115 species belonging to eight orders were collected in the four tributaries (G, Y, R, and L) from 2013 to 2015, which was dramatically less than the 166 species belonging to nine orders recorded during the 1980s (Lu, 1990). Community composition of fish in different tributaries and time periods demonstrated similar characteristics in order taxonomy level (Figure 2), in that the most diverse order was *Cypriniformes,* accounting for 69.56% of the total fish species, followed by *Siluriformes* and *Perciformes* accounting for 13.04% and 12.17%, respectively. The comparison of species composition based on Jaccard's similarity index revealed distinct variations in space and time (Table 1). Firstly, the current similarities values of community composition among tributaries were higher than the results derived from the 1980s, which indicated an emergent trend of increased homogeneity during the more recent past. Secondly, the similarity of community composition between G and Y tributary (0.388 and 0.515) were higher than between R and L tributary (0.526 and 0.663), both in the 1980s and 2013–2015. Finally, the pairwise comparison of community composition between the 1980s and 2013–2015 exhibited a low similarity (from 0.354 to 0.419) in each tributary, which revealed substantial species replacement in each tributary over the past few decades.

**Table 1:** Pairwise comparison of species similarity (Jaccard's index) between tributaries, values in lower left corner corresponding to similarity between tributaries in the 1980s, values in upper right corner corresponding to similarity between tributaries from 2013 to 2015, and the bold values in the center of the table corresponding to similarity between different time periods of each tributary

	G13–15	Y13–15	R13–15	L13–15	
G 80s	**0.354**	0.515	0.586	0.596	G13–15
Y 80s	0.388	**0.402**	0.618	0.611	Y13–15
R 80s	0.436	0.433	**0.402**	0.663	R13–15
L 80s	0.475	0.426	0.526	**0.419**	L13–15
	G 80s	Y 80s	R 80s	L 80s	

G, Y, R, and L represent Guijiang, Yujiang, Youjiang, and Zuojiang, respectively; 13–15 equals to 2013–2015, and 80s equals to the 1980s.

### 3.2 Relationships between physiochemical data and biodiversity indices

**Figure 3:**
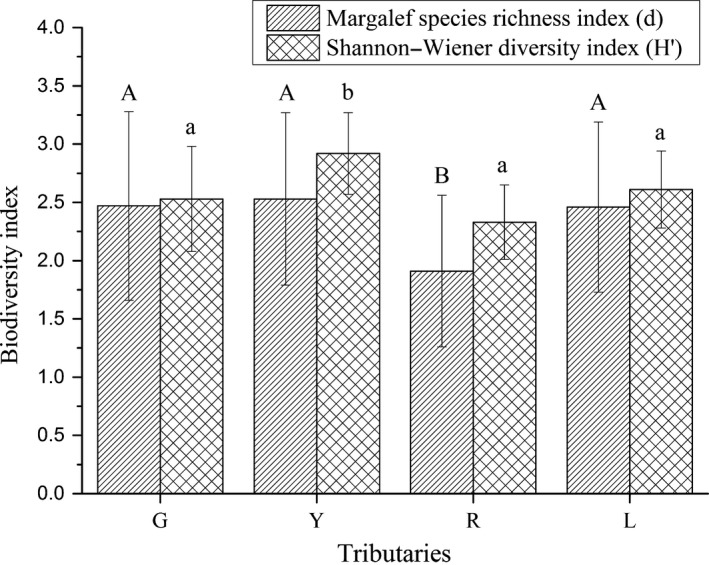
Mean annual values of species richness (*d*) and fish diversity (*H*′) in each tributary (G, Y, R, L) and their spatial variations from 2013 to 2015. Capital letter and lowercase represent the statistical variations of species richness and fish diversity, respectively

Variations of species richness (Figure 3) were noticed among different tributaries ranging from 1.91 (R) to 2.53 (Y). Species richness was significantly lower in R tributary than in all other tributaries (*p* < .001, *p* = .002, and *p* = .001 in G, Y, and Z tributary, respectively). The Shannon–Weiner diversity index varied from 2.33 (R) to 2.72 (Y) in the studied tributaries, and a significantly greater *H*′ score was detected from the Y tributary compared with the other three tributaries (*p* = .018, *p* < .001, and *p* = .001 in G, R, and Z tributary, respectively). As expected, the maximum biodiversity value observed was accompanied with higher species richness in the Y tributary, which indicated relatively less community homogenization than observed in the other tributaries.

Furthermore, three of the selected physiochemical data (pH, DO, and TP) demonstrated significant differences among tributaries (*p* = .014, *p* < .01, and *p* < .01, respectively; Table 2). The mean pH value of 7.47 in G tributary was significantly lower than the result in Y (7.68; *p* = .047), R (7.7; *p* = .016), and L (7.76; *p* = .002) tributaries, and a higher TP concentration of 0.06 mg/L was obtained in Y tributary than other tributaries (*p* < .001, *p* < .001, and *p* = .001 in G, Y, and Z tributary, respectively). DO concentration varied significantly across tributaries (*p* < .001). Statistical correlation among biodiversity indices and selected physiochemical data indicated that TP impacted on species richness positively, while DO showed negative effects (Table 3).

**Table 2:** Mean, standard deviation, and univariate statistical analyses of physiochemical environmental variables by tributary at the 95% confidence level in Guangxi Province from 2013 to 2015

	G	Y	R	L	Test (*F*)	*p*
pH	7.47 ± 0.24	7.68 ± 0.25	7.7 ± 0.4	7.76 ± 0.21	3.824	.014
DO	7.92 ± 1.63	6.18 ± 0.91	7.75 ± 0.78	6.9 ± 0.77	8.316	≤.01
MnO_4_^−^	1.53 ± 0.49	1.92 ± 0.59	2.02 ± 0.74	1.7 ± 0.79	2.027	.118
NH_3_–N	0.1 ± 0.06	0.16 ± 0.13	0.09 ± 0.12	0.11 ± 0.2	0.694	.559
TP	0.03 ± 0.02	0.06 ± 0.03	0.01 ± 0.01	0.02 ± 0.01	27.044	≤.01

G, Y, R, and L represent Guijiang, Yujiang, Youjiang, and Zuojiang, respectively. TP and DO are abbreviations of total phosphorus and dissolved oxygen, respectively.

**Table 3:** The Pearson correlation analysis between water chemistry factors and fish diversity index in different sample sites and seasons at the .01 level in Guangxi Province

	pH	DO	MnO_4_^−^	NH_3_–N	TP	*d*	*H*′
pH	1	−0.49	−0.165	−0.001	−0.199	−0.007	0.09
DO	−0.49	1	0.07	−0.02	−0.313^a^	−0.299^b^	−0.507^a^
MnO_4_^−^	−0.165	0.07	1	0.465^a^	0.002	0.013	0.001
NH_3_–N	−0.001	−0.02	0.465^a^	1	0.18	0.025	−0.03
TP	−0.199	−0.313^a^	0.002	0.18	1	0.244^b^	0.263^b^
*d*	−0.007	−0.299^b^	0.013	0.025	0.244^b^	1	0.845^a^
*H*′	0.09	−0.507^a^	0.001	−0.03	0.263^b^	0.845^a^	1

*d* represents species richness; *H*′ represents Shannon–Wiener diversity. TP and DO are abbreviations of total phosphorus and dissolved oxygen, respectively.

a Correlation is significant at the .01 level (two‐tailed).

b Correlation is significant at the .05 level (two‐tailed).

### 3.3 Spatial heterogeneity of fish community

**Figure 4:**
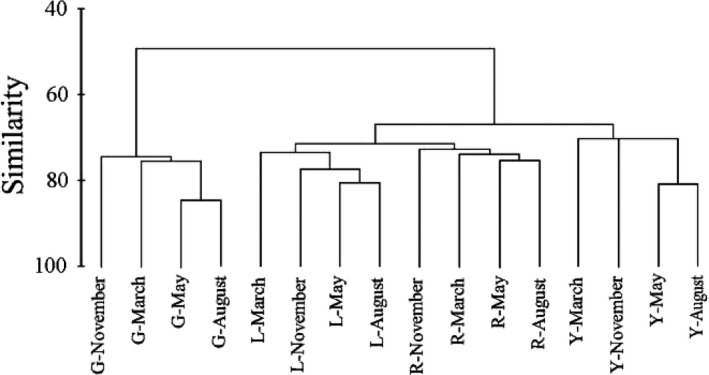
Cluster analysis of seasonal and regional fish community structure based on fish biomass. (G, Y, R, and L represent Guijiang, Yujiang, Youjiang, and Zuojiang respectively)

**Figure 5:**
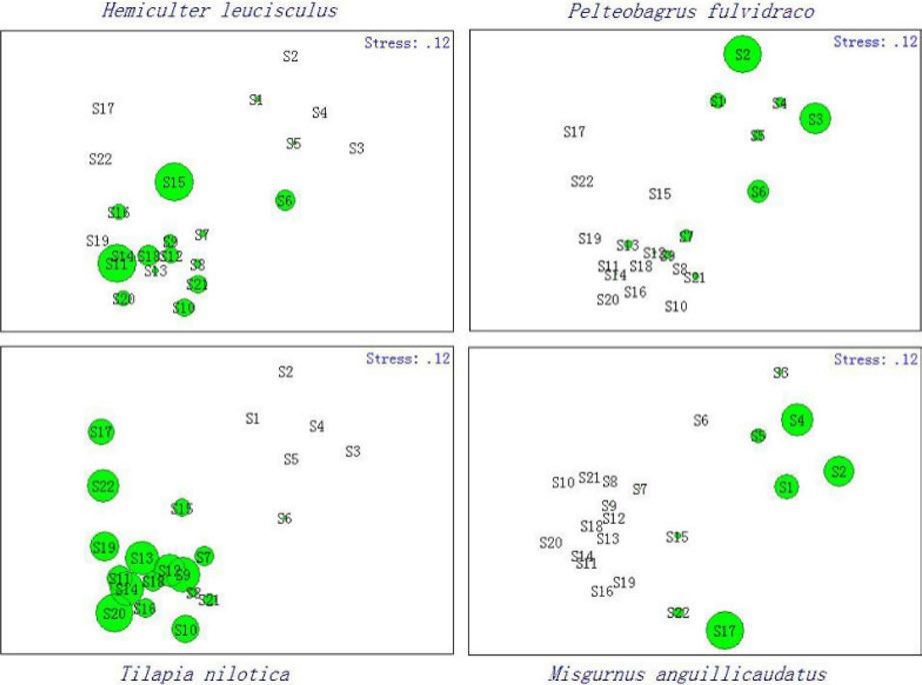
The distribution of four indicative fish species in two eco‐regions (G tributary alone and Y, R, and L combined) according to the similarity percentage procedure analysis on fish assemblage in each tributary from 2013 to 2015 (G, Y, R, and L represent Guijiang, Yujiang, Youjiang, and Zuojiang, respectively; diameter size of each circle was positive to the abundance of the corresponding fish species)

The studied tributaries would be divided into two eco‐regions according to the results from cluster analysis, and the differentiation of fish community intertributaries was much more considerable than interseasons (Figure 4). Accordingly, the first eco‐region (A) only included G tributary and the other three tributaries—Y, R, and L—formed the second eco‐region (B). The MDS analysis corroborated the cluster, providing a two‐dimensional solution with an acceptable stress value of 0.07. ANOSIM revealed differentiation between two eco‐regions (*R* = .926; *p* < .001), which indicated spatial heterogeneity of fish community between G tributary and other tributaries. The SIMPER procedure revealed that community similarity in the six sample sites of eco‐region A and sixteen sample sites of eco‐region B was 49.82% and 53.14%, respectively, and dissimilarity between A and B eco‐regions was 72.43%, which indicated a distinction between the two eco‐regions (Table 4). Species contributing mostly to community similarity inner eco‐regions and dissimilarity inter‐eco‐regions were deemed to biological indicator of different habitats. In this study, *yellow catfish* and *pond loach* were considered to be the indicative species in eco‐region A, and the indicative species in eco‐region B were *N. tilapia* and *sharpbelly* (Table 4). The MDS values plotted in Figure 5 corroborate the SIMPER results.

**Table 4:** Species contribute beyond 5% to similarity inner eco‐regions and dissimilarity between eco‐regions based on similarity percentage procedure analyses in the investigated tributaries in Guangxi Province from 2013 to 2015

	A eco‐region		B eco‐region		A versus B	
	Av. sim		Av. sim		Av. diss	
	49.82		51.34		72.43	
	Sim	Contrib	Sim	Contrib	Diss	Contrib
*Yellow catfish*	12.9	25.9			7.79	10.76
*Pond loach*	8.02	16.1			6.53	9.01
*Common carp*	5	10.03	6.07	11.83		
*Zacco platypus*	3.36	6.75				
*Crucian carp*	3.14	6.3	5.27	10.27		
*Nile tilapia*			12.64	24.63	7.79	10.75
*Sharpbelly*			7.14	13.91	5.25	7.24
*Oreochromis mossambicus*			4.67	9.1		

Av. sim represents average similarity; Av. diss represents average dissimilarity; Contrib represents contribution.

### 3.4 The correlation between fish community and physiochemical environmental factors

**Figure 6:**
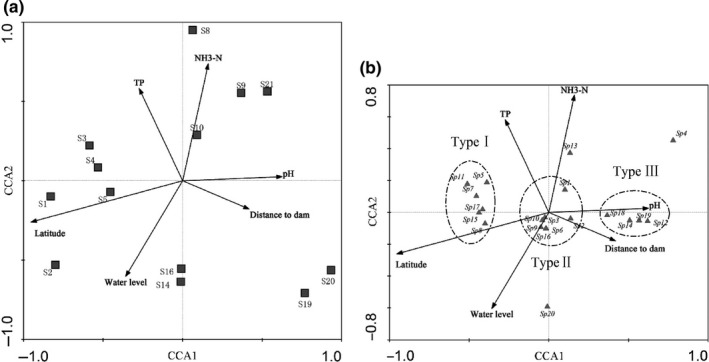
Canonical correspondence analysis biplots describe the relationship between physicochemical environmental factors and sampling sites (a) and fish assemblage of important species (b) in all investigated tributaries from 2013 to 2015. Length and direction of arrows indicate the relative importance and direction of change of the physicochemical environmental factors, respectively. *S*_*i*_ (*i* = 1, 2, 3, 4, 5, 8, 9, 10, 14, 16, 19, 20, 21) represent sampling sites, and Sp1–20 represent the important fish species in all tributaries referring to Appendix

Canonical correspondence analysis ordination scores from an analysis including the relationships between six physiochemical variables and 20 important fish species from 13 sample sites revealed a gradient of three distinct groupings. CCA axes 1 and 2 (CCA1 and CCA2) explained 68.0% and 15.7% of the variance in site‐specific physiochemical variables, respectively (Figure 6a), while CCA1 and CCA2 collectively explained 61.9% of the variance in fish community species composition (Figure 6b). Monte Carlo permutation showed that both axes were significant (*p* = .005). Among the whole physiochemical environmental factors, pH, latitude, and distance to dam were mainly related to CCA1, and the other three factors (water level, TP, and NH_3_–N) were close to CCA2. Vector length of a given variable on the CCA plots indicated the importance of that variable; thus, the factors of pH and latitude contributed more to the spatial variation of fish community when compared to the factor of distance to dam. Distribution of the regional important fish species could be classified into three main groups along the CCA axes (Figure 6b). The first grouping of species had negative scores on CCA1, and these species included those (*yellow catfish*,* pond loach*,* Leiocassis crassilabris, etc*.) that appeared to form an aggregation based on higher latitude sampling sites with lower pH values. The second aggregation (*Tilapia mossambica*,* N. tilapia*,* bighead carp, etc*.) preferred higher pH value and lower latitude was positively correlated with CCA1, mainly distributing along the Y, R, and L tributaries. The remaining fish aggregation (*sharpbelly*,* common carp crucian carp*,* Amur catfish, etc*.) distributed around the center of the biplot, and they were common species which are adaptive to physiochemical variables.

## 4 Discussion

The comparison of fish community composition in different tributaries and time periods demonstrated significant spatial and temporal differentiations. The general declining trend in the number of fish species has reflected several species‐specific conservation threats. In particular, an endemic species named *red stingray* and other two migratory species *Gray's grenadier anchovy Coilia grayii* and *Japanese eel Anguilla japonica* are threatened due to human harvest overexploitation in addition to dam construction and habitat degradation (Morita, Morita, & Yamamoto, 2009; Shaffer et al., 2009). Many other studies have attributed spatial and temporal declines in fish community diversity to the joint actions of human intervention and natural selection (Bunn & Arthington, 2002; Francesco et al., 2010; McKinney, 2006; Olden, 2006; Sapna, Jackson, Minns, & Shuter, 2007; Welcomme, 1985). Specifically, hydropower dam constructions coupled with alien species introductions have been linked to ecological changes and unequal interspecies competition (Kruk & Penczak, 2013; Penczak, 1988; Penczak et al., 2006). These anthropogenic alterations have contributed toward a trend in fish community homogenization throughout multiple watersheds (Brown, 2000; Degerman et al., 2001; McKinney, 2006; Morita, Morita, & Yamamoto, 2009). As for Jaccard's similarity index intertributaries, a typical homogenous trait was presented between the 1980s and 2013–2015 in this study, which was a worldwide trend, coming as a consequence of habitat loss and degradation, and introduction of invasive species (Rahel, 2003).

**Figure 7:**
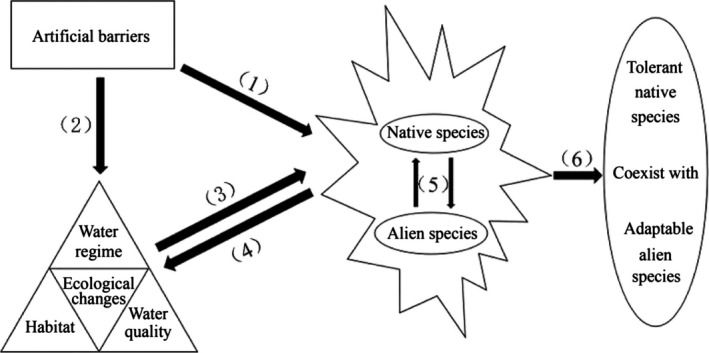
A concise mechanism of interlinking among artificial barriers, ecological changes, and fish assemblage along the investigated freshwater ecosystem over the past few decades in Guangxi Province, China. (1) Artificial barriers block the migration route and restrict the interaction among fish species. (2) Artificial barriers lead to ecological changes which (3) were confirmed to be catastrophic to native fish species impressionable to ecological degradation, in reverse ecological changes fed back on susceptibility to species invasion attributed to changing biodiversity by a variety of mechanisms (Chapin et al., 2000). (4) In return, species invasion accelerate habitat degradation further. (5) The new competition and predator–prey relations were formed between native and alien fish species. (6) After a long period of natural selection and interspecies competition, these tolerant native species and adaptable alien species coexisted in the degraded freshwater ecosystem currently

We propose a conceptual model that helps explain the interlinking mechanisms behind ecological and fish community changes stemming from barrier constructions and human‐introduced species (Figure 7). Artificial obstacles have fragmented the freshwater ecosystem and caused a chain of ecological effects, and some territorial species impressionable to these changes may face sharp declines in abundance, or be gradually eliminated from the fragmented watersheds. Meanwhile, the alien and other adaptable species spread widely to become common species of the investigated tributaries. A few species, such as *yellowcheek carp Elopichthys bambusa, trilobed‐lip barbel Tor zonatus,* and *shuttle‐like carp Luciocyprinus langsoni,* which were once the dominant species in the 1980s (Lu, 1990; Wang, Zhao, & Zhang, 2007), declined sharply or disappeared altogether from our study sites in 2013–2015. As a result, other native or introduced species such as *sharpbelly*,* yellow catfish*,* T. mossambica*, and *N. tilapia* had become the newly established dominant species in recent decades. Analogous results in Degerman's (2001) research have been reported that dam constructions leading to water‐level regulation impeded the migratory activities of diadromous fish species (eel). However, the ecological effects caused by human activities were detrimental to the eel and favorable to the crayfish. As a result, the eel population in L. Hjälmaren Lake declined, and an immense expansion of the noble crayfish population took place.

Accidental and intentional introduction of alien fish species tolerant to watershed fragmentation have been reported to have negative impacts on native fish species in many locations (Degerman et al., 2001; Mills, Rader, & Belk, 2004; Rincon, Correas, Risueno, & Lobon‐Cervia, 2002). *Nile tilapia* and *Oreochromis mossambicus* have been introduced to many countries and regions for aquaculture, owing to their prominent growth properties and superior ability to adapt to novel environments (Liti, Cherop, Munguti, & Chhorn, 2005; Peterson et al., 2006). These species were reported to have invaded a large number of natural water bodies as a result of escaping and breeding outside of designated—but negligently managed—aquaculture areas (Grammer, Slack, Peterson, & Dugo, 2012; Russell, Thuesen, & Thomson, 2012; Zengeya, Robertson, Booth, & Chimimba, 2013). Currently, the population of these species has increased rapidly, and a natural population has established mainly in Guangdong and Guangxi provinces (Gu, Mu, Luo, Li, Wang, et al., 2012; Gu, Mu, Luo, Li, Yang, et al., 2012; Tan, Li, & Li, 2012), resulting in a severe ecological crisis. As a matter of fact, a large amount of native fish species have been replaced or evicted from their original habitats by *N. tilapia* and *O. mossambicus*, which are characterized by competitiveness, aggressiveness, and territorial behavior, when compared to native species (Martin, Valentine, & Valentine, 2010). *Nile tilapia* and *O. mossambicus* are omnivorous species and can disturb the survival and reproductive success of some native species by preying on their fish eggs and larvae (Goudswaard, Witte, & Katunzi, 2002). As a result, the majority of competitive and adaptable alien and native species can coexist and thrive in ecosystems impacted by human development, which has been supported by other studies (Prochelle & Campos, 1985; Rincon et al., 2002). In conclusion, dam construction leading to habit degradation and the unequal predation and competition relationship between native and alien fish species combined to propel the homogenization progress of fish community in the studied tributaries over the past few decades.

Species richness, biodiversity, and physiochemical environmental factors were characterized in concert to elucidate impacts from anthropogenic influences. Species richness in the R tributary was relatively lower than observed in others. DO and TP have been found to be vital environmental factors associated with species richness (César et al., 2012), and our results support this hypothesis. Water level may well be another driver leading to a lower species richness in R tributary, as the mean annual water level in R tributary elevated dramatically over the past few decades. Along with the raise of water level, the physiochemical environmental factors have been changing in different water layers simultaneously, likely making the environmental conditions in the water bed less ideal for demersal fish (such as *trilobed‐lip barbel*) to inhabit. The dominant fish species in R tributary were pelagic fish, such as *sharpbelly*,* Toxabramis houdemeri*, and *N. tilapia*. Biodiversity has been reported to be effected by habitat and food availability owing to water‐level fluctuations (van den Berg et al., 2002). Despite the shortage of previous analysis, the present study would be useful in providing baseline data for any future assessment on fish biodiversity.

In this study, the spatial differentiation of fish community was explained by a few dominant fish species in each classified group (Table 4). The analysis of feeding habits of fishes indicated dominancy of omnivorous fishes in all tributaries. Similar findings as per Fu et al. (2003) and Das and Chakrabarty (2007) were reported from the Yangtze River basin of China and two tropical rivers in India, respectively. Omnivores were often the most tolerant of degraded ecosystems, as they were able to consume food from a wide variety of sources in a changing environment (Wichert & Rapport, 1998). It was expected that fish with a high fecundity and a short reproductive cycle would be able to adapt to the new ecosystem and be impacted less in the long run, while fish with a longer reproductive cycle and low fecundity would suffer greater impacts from the environmental changes (Jennings, Reynolds, & Mills, 1998; Kang et al., 2009). Such a situation was literally exhibited in the investigated tributaries over the past few decades: The once‐dominant carnivorous species, such as *black carp*,* trilobed‐lip barbel*, and *helmet catfish,* were replaced by the omnivorous species *crucian carp*,* common carp*,* N. tilapia,* and others*,* which helps explain the current composition of the dominant species in the investigated tributaries.

It would be hard to expect such large‐scale and long‐term directional changes in the spatial–temporal patterns of species populations without environmental change (Quist, Rahel, & Hubert, 2005). Substrate configuration of riverbed, dissolved oxygen, water temperature, width, depth and flow velocity of river basins have been documented key drivers impacting the distribution of fish communities (Brown, 2000; Kadye et al., 2008; Smith & Kraft, 2005). Therefore, in order to determine which drivers and the extent of those drivers influence on a particular species, we must combine observed changes in drivers with the occurrence, abundance, and traits of dominant species (Pool & Olden, 2011). The present study revealed that the physicochemical habitat variables played an important role in the classification of fish community. Latitude and pH were key habitat features, which correlated with the classification of fish populations in tributaries, allowing the dominant species to be classified into three groups in general. A similar pattern of habitat attributes has been observed by Iii and Orth (1991) and Shahnawaz, Venkateshwarlu, Somashekar, and Santosh (2010). The research by Arroyo et al. (2013) has discovered that β‐diversity among patches was more related to the geographical distance between sampled patches than other factors and higher β‐diversity was obtained between sample patches more isolated which explained the spatial differentiation of fish community well between G and other three tributaries. Other factors such as NH_3_–N, TP, and water level are known to be important in the physiological behavior of fish (Fraser, 1997) and contributed relatively less than geographical distance to the classification of fish populations in the studied physical area.

Physicochemical habitat variables, construction of hydropower dams, and introduction of alien fish species can push the spatial differentiation of fish communities to some extent by direct or indirect processes. On the one hand, artificial barriers—especially cascade dams—in natural freshwater ecosystems primarily break the route of migratory fishes. This restricts the distribution of these species and can result in the spatial heterogeneity of fish community (Baran, 2006). In particular, dams in the investigated tributaries were generally constructed without appropriate fish ways or fish passes, except Laokou and Yuliang hydrojunction, which amplified the effects of dam projects on migratory fish species. Dam constructions can regulate water level, alter flow velocity, and transform habitat types which would change the spatial structure of fish community, albeit in an indirect manner (Salazar, 2000). The naturalization and range expansion of alien fish species would restrain the development of some native species in certain tributaries. There are many examples of large‐scale and dramatic effects of alien species on indigenous species (e.g., *Nile perch*,* Lates niloticus*, in Lake Victoria, the *crayfish plague* in Europe, *salmonids* in Southern Hemisphere lakes and streams; see Rahel, 2003). Nonnatives have been well documented to contribute either to the homogeneity or to the heterogeneity of fish populations (Marchetti, Lockwood, & Light, 2006; Olden, Poff, & McKinney, 2006). In this study, the explosive enhancement of *O. mossambicus* and *N. tilapia* in Y, R, and L tributaries may well be a promoter of spatial heterogeneity of fish community. Widespread invasion and deliberate introduction of alien species as a result of human activity can have indirect effects on native species, as alien species are most likely to successfully invade freshwaters already modified or degraded by humans (e.g., Bunn & Arthington, 2002; Koehn, 2004).

In conclusion, these novel findings showed that fish communities can undergo a homogeneous/heterogeneous trend in the fragmented riverine ecosystem attributing to human activities such as dam construction, introduction of alien fish species, and habitat degradation over the past few decades. Understanding the shifting distributions of fish communities in space and time and what anthropogenic and natural ecological processes contribute to these changes is necessary to help develop an appropriate management policy. Factors involved in this research were limited in order to explain the homogeneity/heterogeneity of fish communities in a precise manner. To make it more compelling, other factors related to dam construction, such as flow velocity and water temperature, should be taken into account in further studies.

## Conflict of Interest

None declared.

## Appendix 1 Regional important fish species

### 1.1

**Table:**

Species name	Symbol	Region type	Environmental property
*Leiocassis crassilabris*	sp5	Type I	Higher latitude and lower pH
*Pelteobagrus fulvidraco*	sp7	Type I	Higher latitude and lower pH
*Monopterus albus*	sp8	Type I	Higher latitude and lower pH
*Opsariichthys bidens*	sp11	Type I	Higher latitude and lower pH
*Misgurnus anguillicaudatus*	sp15	Type I	Higher latitude and lower pH
*Pelteobaggrus vachelli*	sp17	Type I	Higher latitude and lower pH
*Mystus guttatus*	sp1	Type II	Eurytopic
*Hemiculter leucisculus*	sp2	Type II	Eurytopic
*Ctenopharyngodon idellus*	sp3	Type II	Eurytopic
*Clarias fuscus*	sp6	Type II	Eurytopic
*Carassius auratus*	sp9	Type II	Eurytopic
*Cyprinus carpio*	sp10	Type II	Eurytopic
*Pseudohemiculter dispar*	sp13	Type II	Eurytopic
*Silurus asotus*	sp16	Type II	Eurytopic
*Rhinogobius giurinus*	sp20	Type II	Eurytopic
*Spualiobarbus curriculus*	sp4	Type III	Higher pH and lower latitude
*Oreochromis mossambicus*	sp12	Type III	Higher pH and lower latitude
*Tilapia nilotica*	sp14	Type III	Higher pH and lower latitude
*Squalidus argentatus*	sp18	Type III	Higher pH and lower latitude
*Aristichthys nobilis*	sp19	Type III	Higher pH and lower latitude

## References

[ece32920-bib-0001] Allan, J. D. , & Flecker, A. S. (1993). Biodiversity conservation in running waters. BioScience, 43, 32–43.

[ece32920-bib-0002] Arroyo, R. V. , Rös, M. , Escobar, F. , Melo, F. P. L. , Santos, B. A. , Tabarelli, M. , & Chazdon, R. (2013). Plant β‐diversity in fragmented rain forests: Testing floristic homogenization and differentiation hypotheses. Journal of Ecology, 101, 1449–1458.

[ece32920-bib-0003] Baran, E. (2006). Fish migration triggers in the Lower Mekong basin and other tropical freshwater systems. MRC Technical Paper No. 14. Mekong River Commission, Vientiane, Laos.

[ece32920-bib-0004] Braak, C. J. F. T. (1986). Canonical correspondence analysis: A new eigenvector technique for multivariate direct gradient analysis. Ecology, 67, 1167–1179.

[ece32920-bib-0005] Bray, J. R. , & Curtis, J. T. (1957). An ordination of the upland forest communities of southern Wisconsin. Ecological Monographs, 27, 325–349.

[ece32920-bib-0006] Brown, L. R. (2000). Fish communities and their associations with environmental variables, lower San Joaquin River drainage, California. Environmental Biology of Fishes, 57, 251–269.

[ece32920-bib-0007] Bunn, S. E. , & Arthington, A. H. (2002). Basic principles and ecological consequences of altered flow regimes for aquatic biodiversity. Environmental Management, 30, 492–507. 12481916 10.1007/s00267-002-2737-0

[ece32920-bib-0008] Cadotte, M. W. (2011). The new diversity: Management gains through insights into the functional diversity of communities. Journal of Applied Ecology, 48, 1067–1069.

[ece32920-bib-0009] César, I. I. , Martín, S. M. , Rumi, A. , & Tassara, M. (2012). Mollusks (Gastropoda and Bivalvia) of the multiple‐use reserve Martín García Island, Río de la Plata River: Biodiversity and ecology. Brazilian Journal of Biology, 72(1), 121–130. 10.1590/s1519-69842012000100014 22437392

[ece32920-bib-0010] Chapin, F. S. , Zavaleta, E. S. , Eviner, V. T. , Naylor, R. , Vitousek, P. M. , Reynolds, H. L. , … Diaz, S. (2000). Consequences of changing biodiversity. Nature, 405(6783), 234–242. 10821284 10.1038/35012241

[ece32920-bib-0012] Clarke, K. R. , & Gorley, R. N. (2001). PRIMER v5: User manual/tutorial. Plymouth, UK: PRIMER‐E.

[ece32920-bib-0013] Crist, T. O. , & Veech, J. A. (2006). Additive partitioning of rarefaction curves and species‐area relationships: Unifying alpha‐, beta‐ and gamma‐diversity with sample size and habitat area. Ecology Letters, 9, 923–932. 16913935 10.1111/j.1461-0248.2006.00941.x

[ece32920-bib-0014] Das, S. K. , & Chakrabarty, D. (2007). The use of fish community structure as a measure of ecological degradation: A case study in two tropical rivers of India. Biosystems, 90, 188–196. 17023110 10.1016/j.biosystems.2006.08.003

[ece32920-bib-0015] Degerman, E. , Hammar, J. , Nyberg, P. , et al. (2001). Human impact on the fish diversity in the four largest lakes of Sweden. Journal of the Human Environment, 30, 522–528. 10.1579/0044-7447-30.8.522 11878026

[ece32920-bib-0016] Dudgeon, D. (2000). Large‐scale hydrological changes in tropical Asia: Prospects for riverine biodiversity. BioScience, 50, 793–806.

[ece32920-bib-0017] Dudgeon, D. , & Smith, R. E. W. (2006). Exotic species, fisheries and conservation of freshwater biodiversity in tropical Asia: The case of the Sepik River, Papua New Guinea. Aquatic Conservation Marine and Freshwater Ecosystems, 16, 203–215.

[ece32920-bib-0018] Dynesius, M. , & Nilsson, C. (1994). Fragmentation and flow regulation of river systems in the northern third the world. Science, 266, 753–762. 17730396 10.1126/science.266.5186.753

[ece32920-bib-0019] Fausch, K. D. , Lyons, J. , Karr, J. R. , & Angermeier, P. L. (1990). Fish communities as indicators of environmental degradation. American Fisheries Society Symposium, 8, 123–144.

[ece32920-bib-0020] Fortin, M. J. , & Gurevitch, J. (2001). Mantel tests. Spatial structure in field experiments In Scheiner S. M. & Gurevitch J. (Eds.), Design and analysis of ecological experiments (2nd ed., pp. 308–326). Oxford, UK: Oxford University Press.

[ece32920-bib-0021] Francesco, F. G. , Luigi, M. , Alessandra, F. , Nicolas, D. , Luigi, B. , Emilio, P. S. , … Wilfried, T. (2010). Knowing the past to predict the future: Land‐use change and the distribution of invasive bullfrogs. Global Change Biology, 16, 528–537.

[ece32920-bib-0022] Fraser, T. H. (1997). Abundance, seasonality, community indices, trends and relationships with physicochemical factors of trawled fish in upper Charlotte Harbor, Florida. Bulletin of Marine Science, 60, 739–763.

[ece32920-bib-0023] Freeman, R. , Bowerman, W. , Grubb, T. , Bath, A. , Dawson, G. , Ennis, K. , & Giesy, J. (2002). Opening rivers to Trojan fish: The ecological dilemma of dam removal in the Great Lakes. Conservation in Practice, 3, 35–40.

[ece32920-bib-0024] Fu, C. , Wu, J. , Chen, J. , Wu, Q. , & Lei, G. (2003). Freshwater fish biodiversity in the Yangtze River basin of China: Patterns, threats and conservation. Biodiversity and Conservation, 12, 1649–1685.

[ece32920-bib-0025] Goudswaard, P. C. , Witte, F. , & Katunzi, E. F. B. (2002). The tilapiine fish stock of Lake Victoria before and after the Nile perch upsurge. Journal of Fish Biology, 60, 838–856.

[ece32920-bib-0026] Grammer, G. L. , Slack, W. T. , Peterson, M. S. , & Dugo, M. A. (2012). Nile tilapia *Oreochromis niloticus* (Linnaeus, 1758) establishment in temperate Mississippi, USA: Multi‐year survival confirmed by otolith ages. Aquatic Invasions, 7, 367–376.

[ece32920-bib-0027] Gu, D. E. , Mu, X. D. , Luo, D. , Li, Y. Y. , Wang, X. J. , Song, H. M. , … Hu, Y. C. (2012). The distribution of alien aquatic animals in the main rivers of Guangdong Province, China. Journal of Biosafety, 21, 272–276.

[ece32920-bib-0028] Gu, D. E. , Mu, X. D. , Luo, D. , Li, Y. Y. , Yang, Y. X. , Xu, M. , & Hu, Y. C. (2012). The study of population establishment of tilapia in main rivers in Guangdong Province, China. Journal of Biosafety, 21, 277–282.

[ece32920-bib-0029] He, D. M. , Li, S. , & Zhang, Y. P. (2007). The variation and regional differences of precipitation in the Longitudinal Range‐Gorge the Region. Chinese Science Bulletin, 52, 59–73.

[ece32920-bib-0030] Iii, M. D. L. , & Orth, D. J. (1991). Habitat use by an assemblage of fish in a large warm water stream. Transactions of the American Fisheries Society, 120, 65–78.

[ece32920-bib-0031] Jackson, R. B. , & Running, S. W. (2001). Water in a changing world. Ecological Applications, 11, 1027–1045.

[ece32920-bib-0032] Jennings, S. , Reynolds, J. D. , & Mills, S. C. (1998). Life history correlates of responses to fisheries exploitation. Proceedings of the Royal Society of London, Series B: Biological Sciences, 265, 1–7.9470212

[ece32920-bib-0033] Kadye, W. T. , Magadza, C. H. D. , Moyo, N. A. G. , et al. (2008). Stream fish assemblages in relation to environmental factors on a montane plateau (Nyika Plateau, Malawi). Environmental Biology of Fishes, 83, 417–428.

[ece32920-bib-0034] Kang, B. , He, D. , Perrett, L. , Wang, H. , Hu, W. , Deng, W. , & Wu, Y. (2009). Fish and fisheries in the Upper Mekong: Current assessment of the fish community, threats and conservation. Reviews in Fish Biology and Fisheries, 19, 465–480.

[ece32920-bib-0035] Koehn, J. D. (2004). Carp (*Cyprinus carpio*) as a powerful invader in Australian waterways. Freshwater Biology, 49, 882–894.

[ece32920-bib-0036] Kruk, A. , & Penczak, T. (2013). Natural regeneration of fish assemblages in the Pilica River after a reduction of point‐source pollution. River Research & Applications, 29, 502–511.

[ece32920-bib-0037] Lever, C. (1996). Naturalized fishes of the world (408 pp.). London, UK: Academic Press.

[ece32920-bib-0038] Liti, D. , Cherop, L. , Munguti, J. , & Chhorn, L. (2005). Growth and economic performance of Nile tilapia (*Oreochromis niloticus* L.) fed on two formulated diets and two locally available feeds in fertilized ponds. Aquaculture Research, 36, 746–752.

[ece32920-bib-0039] Liu, J. (1984). Lakes of middle and lower basins of Changjiang (China) In Taub F. B. (Ed.), Lakes and reservoirs (pp. 331–355). Amsterdam, the Netherlands: Elsevier.

[ece32920-bib-0040] Liu, J. (1992). Fish resources of the Yangtze River basin and the tactics for their conservation. Resources & Environment in the Yangtze Basin, 1, 17–23.

[ece32920-bib-0041] Loreau, M. (2000). Are communities saturated? On the relationship between alpha, beta and gamma diversity. Ecology Letters, 3, 73–76.

[ece32920-bib-0042] Lu, K. X. (1990). Fishery resource of Pearl River. Guangzhou, China: Guangdong Science and Technology Press.

[ece32920-bib-0043] Lundberg, J. G. , & Gill, A. C. (2000). So many fishes, so little time: An overview of recent ichthyological discovery in continental waters. Annals of the Missouri Botanical Garden, 87, 26–62.

[ece32920-bib-0045] Malmqvist, B. , & Rundle, S. (2002). Threats to the running water ecosystems of the world. Environmental Conservation, 29, 134–153.

[ece32920-bib-0046] Marchetti, M. P. , Lockwood, J. L. , & Light, T. (2006). Effects of urbanization on California's fish diversity: Differentiation, homogenization and the influence of spatial scale. Biological Conservation, 127, 310–318.

[ece32920-bib-0047] Martin, C. W. , Valentine, M. M. , & Valentine, J. F. (2010). Competitive interactions between invasive Nile tilapia and native fish: The potential for altered trophic exchange and modification of food webs. PLoS One, 5, e14395. 21200433 10.1371/journal.pone.0014395 PMC3006172

[ece32920-bib-0048] McCormick, F. H. , & Larsen, D. P. (2000). Comparison of geographic classification schemes for Mid‐Atlantic stream fish assemblages. Journal of the North American Benthological Society, 19, 385–404.

[ece32920-bib-0049] McKinney, M. L. (2006). Urbanization as a major cause of biotic homogenization. Biological Conservation, 127, 247–260.

[ece32920-bib-0050] McKinney, M. L. , & Lockwood, J. L. (1999). Biotic homogenization: A few winners replacing many losers in the next mass extinction. Trends in Ecology and Evolution, 14, 450–453. 10511724 10.1016/s0169-5347(99)01679-1

[ece32920-bib-0051] Mills, M. D. , Rader, R. B. , & Belk, M. C. (2004). Complex interactions between native and invasive fish: The simultaneous effects of multiple negative interactions. Oecologia, 141, 713–721. 15322899 10.1007/s00442-004-1695-z

[ece32920-bib-0052] Morita, K. , Morita, S. H. , & Yamamoto, S. (2009). Effects of habitat fragmentation by damming on salmonid fishes: Lessons from white‐spotted charr in Japan. Ecological Research, 24, 711–722.

[ece32920-bib-0053] Naiman, R. J. , & Turner, M. G. (2000). A future perspective on North America's freshwater ecosystems. Ecological Applications, 10, 958–970.

[ece32920-bib-0054] Olden, J. (2006). Biotic homogenization: A new research agenda for conservation biogeography. Journal of Biogeography, 33, 2027–2039.

[ece32920-bib-0055] Olden, J. D. , Poff, L. R. , & McKinney, M. L. (2006). Forecasting faunal and floral homogenization associated with human population geography in North America. Biological Conservation, 127, 261–271.

[ece32920-bib-0056] Penczak, T. (1988). The ichthyofauna of the Pilica drainage basin. Part I. Pre‐ impoundment study. Scientific Annual of the Polish Angling Association, 1, 23–59.

[ece32920-bib-0057] Penczak, T. , Kruk, A. , Ziezba, G. , Marszał, L. , Koszalinski, H. , Tybulczuk, S. , & Galicka, W. (2006). Fish fauna in the Pilica River system in the fifth decade of study. Part I. Pilica River. Scientific Annual of the Polish Angling Association, 19, 103–122.

[ece32920-bib-0058] Peterson, M. S. , Slack, W. T. , Waggy, G. L. , Finley, J. , Woodley, C. M. , & Partyka, M. L. (2006). Foraging in non‐native environments: Comparison of Nile tilapia and three co‐occurring native centrarchids in invaded Coastal Mississippi Watersheds. Environmental Biology of Fishes, 76, 283–301.

[ece32920-bib-0059] Pool, T. K. , & Olden, J. D. (2011). Taxonomic and functional homogenization of an endemic desert fish fauna. Diversity and Distributions, 18, 366–376.

[ece32920-bib-0060] Prochelle, O. , & Campos, H. (1985). The biology of the introduced carp *Cyprinus carpio* L., in the river Cayumapu, Valdivia, Chile. Studies on Neotropical Fauna and Environment, 20, 65–82.

[ece32920-bib-0061] Quist, M. C. , Rahel, F. J. , & Hubert, W. A. (2005). Hierarchical faunal filters: An approach to assessing effects of habitat and nonnative species on native fishes. Ecology of Freshwater Fish, 14, 24–39.

[ece32920-bib-0062] Raghavan, R. , Prasad, G. , Anvar‐Ali, P. H. , & Pereira, B. (2008). Exotic fish species in a global biodiversity hot spot: Observations from River Chalakudy, part of Western Ghats, Kerala, India. Biological Invasions, 10, 37–40.

[ece32920-bib-0063] Rahel, F. J. (2003). Homogenization of freshwater faunas. Annual Review of Ecology & Systematics, 33, 291–315.

[ece32920-bib-0065] Rincon, P. A. , Correas, A. F. , Risueno, P. , & Lobon‐Cervia, J. (2002). Interaction between the introduced eastern mosquitofish and two autochthonous Spanish toothcarps. Journal of Fish Biology, 61, 1560–1585.

[ece32920-bib-0066] Russell, D. J. , Thuesen, P. A. , & Thomson, F. E. (2012). A review of the biology, ecology, distribution and control of Mozambique tilapia, *Oreochromis mossambicus* (Peters 1852) (Pisces: Cichlidae) with particular emphasis on invasive Australian populations. Reviews in Fish Biology and Fisheries, 22, 533–554.

[ece32920-bib-0067] Salazar, J. G. (2000). Damming the child of the ocean: The three gorges project. Journal of Environment & Development, 9, 160–174.

[ece32920-bib-0068] Sapna, S. , Jackson, D. A. , Minns, C. K. , & Shuter, B. J. (2007). Will northern fish populations be in hot water because of climate change? Global Change Biology, 13, 2052–2064.

[ece32920-bib-0069] Shaffer, J. A. , Beirne, M. , Ritchie, T. , Paradis, R. , Barry, D. , & Crain, P. (2009). Fish habitat use response to anthropogenic induced changes of physical processes in the Elwha estuary, Washington, USA. Hydrobiologia, 636, 179–190.

[ece32920-bib-0070] Shahnawaz, A. , Venkateshwarlu, M. , Somashekar, D. S. , & Santosh, K. (2010). Fish diversity with relation to water quality of Bhadra River of Western Ghats (INDIA). Environmental Monitoring and Assessment, 161, 83–91. 19184486 10.1007/s10661-008-0729-0

[ece32920-bib-0071] Simon, K. S. , & Townsend, C. R. (2003). Impacts of freshwater invaders at different levels of ecological organisation, with emphasis on salmonids and ecosystem consequences. Freshwater Biology, 48, 982–994.

[ece32920-bib-0072] Smith, T. A. , & Kraft, C. E. (2005). Stream fish assemblages in relation to landscape position and local habitat variables. Transactions of the American Fisheries, 134, 430–440.

[ece32920-bib-0073] Tan, Z. Y. (2012). The study of Tilapia industry development situation in Guangxi. PhD Thesis, Guangxi University, Nanning, China.

[ece32920-bib-0074] Tan, X. C. , Li, X. H. , & Li, Y. F. (2012). Early development and spatial distribution of the Nile tilapia (*Oreochromis niloticus*) in the Pearl River. Journal of Biosafety, 21, 295–299.

[ece32920-bib-0075] Taylor, E. B. (2010). Changes in taxonomy and species distributions and their influence on estimates of faunal homogenization and differentiation in freshwater fishes. Diversity and Distributions, 16, 676–689.

[ece32920-bib-0076] Taylor, J. N. , Courtenay, W. R. , & McCann, J. A. (1984). Known impacts of exotic fishes in the continental United States Distribution, biology and management of exotic fishes (pp. 322–373). Baltimore, MD: John Hopkins University Press.

[ece32920-bib-0077] van den Berg, M. S. , Broersen, K. W. , Coops, H. , Gotje, W. , Graveland, J. , Haas, H. A. , … Zwarts, L. (2002). Ecologische effecten van peilbeheer: een kennisoverzicht.

[ece32920-bib-0078] Vitousek, P. M. , Mooney, H. A. , Lubchenco, J. , & Melillo, J. M. (1997). Human domination of Earth's ecosystems. Science, 277, 494–499.

[ece32920-bib-0079] Wang, D. , Zhao, Y. H. , & Zhang, C. G. (2007). Species diversity of wild freshwater fishes and sustainable utilization of the fish resource in Guangxi, China. Acta Zootaxonomica Sinica, 32, 160–173.

[ece32920-bib-0080] Welcomme, R. L. (1985). River fisheries. *FAO Fisheries Technical Paper 262* (pp. 1–318). Rome, Italy: Fisheries Department, Food & Agriculture Organization (FAO) of the United Nations.

[ece32920-bib-0501] Welcomme, R. L. (1988). International introductions of inland aquatic species. FAO, UN, Rome

[ece32920-bib-0081] Wellborn, G. A. , Skelly, D. K. , & Werner, E. E. (1996). Mechanisms creating community structure across a freshwater habitat gradient. Annual Review of Ecology and Systematics, 27, 337–363.

[ece32920-bib-0082] Wichert, G. A. , & Rapport, D. J. (1998). Fish community structure as a measure of degradation and rehabilitation of riparian systems in an agricultural drainage basin. Environmental Management, 22, 425–443. 9516535 10.1007/s002679900117

[ece32920-bib-0083] Wilhm, J. L. (1968). Use of biomass units in Shannon's formula. Ecology, 49, 153–156.

[ece32920-bib-0084] Xie, Y. , Li, Z. , Gregg, W. P. , & Li, D. (2001). Invasive species in China—An overview. Biodiversity and Conservation, 10, 1317–1341.

[ece32920-bib-0085] Zeng, X. (1990). Fishery resources of the Yangtze River Basin. Beijing, China: Marine Press.

[ece32920-bib-0086] Zengeya, T. A. , Robertson, M. P. , Booth, A. J. , & Chimimba, C. T. (2013). Ecological niche modeling of the invasive potential of Nile tilapia *Oreochromis niloticus* in African river systems: Concerns and implications for the conservation of indigenous congenerics. Biological Invasions, 15, 1507–1521.

[ece32920-bib-0087] Zhang, H. (2007). The ecological characteristics of fish communities in the Yangtze estuarine wetlands, China. PhD Thesis, East China Normal University, Shanghai, China.

[ece32920-bib-0088] Zhang, G. , Cao, W. , & Chen, Y. (1997). Effects of fish stocking on lake ecosystems in China. Acta Hydrobiologica Sinica, 21, 271–280.

